# Protective interplay: *Mycobacterium tuberculosis* diminishes SARS-CoV-2 severity through innate immune priming

**DOI:** 10.3389/fimmu.2024.1424374

**Published:** 2024-06-20

**Authors:** Brittany D. Williams, Debora Ferede, Hazem F. M. Abdelaal, Bryan J. Berube, Brendan K. Podell, Sasha E. Larsen, Susan L. Baldwin, Rhea N. Coler

**Affiliations:** ^1^ Department of Global Health, University of Washington, Seattle, WA, United States; ^2^ Seattle Children’s Research Institute, Center for Global Infectious Disease Research, Seattle Children’s, Seattle, WA, United States; ^3^ HDT Bio Corp, Seattle, WA, United States; ^4^ Mycobacteria Research Laboratories, Department of Microbiology, Immunology, and Pathology, Colorado State University, Fort Collins, CO, United States; ^5^ Department of Pediatrics, University of Washington School of Medicine, Seattle, WA, United States

**Keywords:** SARS-CoV-2, Mycobacterium tuberculosis, tuberculosis, co-infection, COVID-19

## Abstract

At the beginning of the COVID-19 pandemic those with underlying chronic lung conditions, including tuberculosis (TB), were hypothesized to be at higher risk of severe COVID-19 disease. However, there is inconclusive clinical and preclinical data to confirm the specific risk SARS-CoV-2 poses for the millions of individuals infected with *Mycobacterium tuberculosis* (M.tb). We and others have found that compared to singly infected mice, mice co-infected with M.tb and SARS-CoV-2 leads to reduced SARS-CoV-2 severity compared to mice infected with SARS-CoV-2 alone. Consequently, there is a large interest in identifying the molecular mechanisms responsible for the reduced SARS-CoV-2 infection severity observed in M.tb and SARS-CoV-2 co-infection. To address this, we conducted a comprehensive characterization of a co-infection model and performed mechanistic *in vitro* modeling to dynamically assess how the innate immune response induced by M.tb restricts viral replication. Our study has successfully identified several cytokines that induce the upregulation of anti-viral genes in lung epithelial cells, thereby providing protection prior to challenge with SARS-CoV-2. In conclusion, our study offers a comprehensive understanding of the key pathways induced by an existing bacterial infection that effectively restricts SARS-CoV-2 activity and identifies candidate therapeutic targets for SARS-CoV-2 infection.

## Introduction

The coronavirus disease 2019 (COVID-19), caused by severe acute respiratory syndrome coronavirus 2 (SARS-CoV-2), has resulted in a global pandemic that has claimed over 6.8 million lives as of March 2024 (1). Initially, individuals with underlying chronic lung conditions, including tuberculosis (TB), were thought to be at higher risk of severe COVID-19 and acute respiratory distress syndrome (ARDS) (2). This was a great concern for the 10 million individuals diagnosed with TB in 2019 (3). To speak to its detriment, TB was the long-standing number one infectious disease killer until the start of the COVID-19 pandemic (3). Although the prevalence of COVID-19 and TB co-infection has not been officially confirmed, a recent meta-analysis of 18 studies estimated that the prevalence of TB among COVID-19 positive patients was 1.1% in America, 1.5% in Asia and 3.6% in Africa (4). While TB was later removed as a significant risk factor, conclusive data on the specific risk SARS-CoV-2 poses for the millions infected with *Mycobacterium tuberculosis* (M.tb) remains elusive. Early clinical reports presented conflicting findings with some noting that TB was not a major determinant of mortality (2, 5, 6) and others suggesting co-infection led to worsened outcomes of COVID-19 (7, 8). Additionally, a longitudinal global cohort study which found survival was lower among co-infected individuals discovered certain risk factors, such as age, HIV co-infection, male sex, and invasive ventilation, influenced adverse TB and COVID-19 outcomes (9). Therefore, highlighting multiple factors that may contribute to an individual’s response to SARS-CoV-2 and M.tb infection. While the characterization of the immune response within co-infected individuals has also been limited, studies have reported both overlapping and distinct immune responses (10–12). A clinical study characterizing plasma immune profiles of individuals with TB and COVID-19 versus singular TB or COVID-19 discovered an immune signature composed of TNF-α, MIP-1β, and IL-9 that discriminated co-infection from COVID-19 alone (11). In addition, a signature of TNF-α, IL-1β, IL-17A, IL-5, fibroblast growth factor-basic, and granulocyte macrophage colony-stimulating factor (GM-CSF), has discerned co-infected individuals from those with TB only (11). Indeed, there seems to be a nuanced relationship between M.tb infection and SARS-CoV-2 and multiple demographic and clinical factors may alter the immune response to both infections (2). Understanding how these two pulmonary pathogens interact starts with examining their individual induced innate immune responses, as these responses represent the first line of defense against pathogens.

Primary infection of angiotensin converting enzyme-2 (ACE2)-expressing airway and alveolar epithelial cells by SARS-CoV-2 initiates viral replication, pyroptosis of host cells, and activation of innate immune pathways (13). The innate immune response when properly activated is crucial in providing protection against early SARS-CoV-2 infection. Several pattern recognition receptors (PRRs) detect SARS-CoV-2 and initiate innate responses, including endosomal toll-like receptor 3 (TLR3) and toll-like receptor 7 (TLR7) signaling pathways, as well as cytoplasmic RNA sensor, melanoma differentiation-associated protein 5 (MDA5) (14–16). The cytoplasmic RNA sensor, retinoic acid-inducible gene I (RIG-I), acts more as a restriction factor in which RIG-I detection of the SARS-CoV-2 genome hinders the virus’s first step of replication. Furthermore, knock out of RIG-I was shown to enhance viral activity and virus restriction was rescued with upregulation of RIG-I expression (17). Upon activation of the PRRs, downstream signaling results in the production of antiviral interferons (IFNs), and cytokines and chemokines which create an anti-viral environment and recruits innate cells to the site of infection (18). Type I, II, and III IFNs have been heavily focused on due to their ability to inhibit SARS-CoV-2 replication (19–23). IFN antiviral activity is driven by the upregulation of interferon-stimulated genes (ISGs), which have multiple mechanisms in restricting viral activity (24–26). Multiple ISGs which broadly act against SARS-CoV-2 by inhibiting viral entry, viral RNA synthesis, and virion assembly, have also been identified (26).

However, SARS-CoV-2 has evolved multiple strategies to evade initial innate immune responses, including blocking recognition by host sensors such as RIG-I, MDA5, and TLRs and inhibiting IFN signaling, thus promoting viral replication (27–29). This immune evasion is thought to delay immune responses, leading to unchecked viral replication, high viral load, and a subsequent dysregulated immune response. The resulting disproportionate response to SARS-CoV-2 infection is characterized by a robust release of proinflammatory cytokines and dysfunctional myeloid responses, including elevated levels of IL-2, IL-6, IL-7, IL-10, IL-12, and IL-1β, TNF-α, MCP-1α, IP-10, lymphopenia, and high lung infiltration of monocytes and T cells (13, 30–32).

In contrast to the acute hyperinflammatory profile associated with SARS-CoV-2, chronic M.tb infection is known to elicit a diverse array of proinflammatory and regulatory responses (33). Following initial infection, alveolar macrophages engulf M.tb bacilli, migrate to the lung parenchyma, and orchestrate the recruitment of various innate immune cells and effector T cells. While some infections resolve, others go on to result in granuloma formation, an attempt at prolonged containment by the host and persistent but quiescent latent infection (34–37). Major cell types involved in the control of M.tb are pro-inflammatory T helper 1 (Th1) and Th17 CD4+ T cells which are largely recruited to form a lymphocytic cuff around a core granuloma structure containing macrophages and bacteria. Th1 and Th17 CD4+ T cells express IL-2, IFN-γ, and TNF-a, or IL-17A, IL-21, and IL-23, respectively, which play critical roles in driving immune activation and inflammatory responses designed to control M.tb (33, 38). However certain hallmark stages of granuloma formation and persistent infection include the expression of anti-inflammatory cytokines IL-10, IL-27 and TGF-β to regulate T cell pro-inflammatory activity (33, 39). This balance of immune responses enables local containment of M.tb bacilli without more systemic inflammatory damage.

Further insights can be gleaned from other bacterial and viral co-infection studies. For instance, administering a live-attenuated mycobacteria, bacille Calmette-Guérin (BCG), intravenously, but not subcutaneously, significantly protected mice from SARS-CoV-2 challenge, characterized by reduced lung inflammation and viral burden (40). Similarly, aerosolized exposure to nontypeable *Haemophilus influenzae* (NTHi) bacterial lysate before influenza A infection conferred protection, as evidenced by heightened inflammatory cytokines, decreased viral loads, and increased survival rates in treated mice (41). Notably, while both bacterial exposures provided protection against secondary viral infections, they triggered distinct immune responses, likely influenced by the route of administration, bacterial species and specific PRR pathways induced. These findings collectively underscore the role of nonspecific immune responses in defending against subsequent heterologous infections (42).

Given the global impact of the pandemic, delay of vaccine deployment in many TB endemic low- and middle-income countries (LMICs), and continuous emergence of hyper-transmissible variants, it is unknown how long the pandemic and its ramifications will last. This highlights the need to study co-infections to identify disease burdens, mechanisms of immunopathology, and heterologous protection to better inform susceptibility and population risk. With our work, we tested our hypothesis that acute M.tb infection induces a diffuse innate immune response within the lungs leading to a primed lung epithelium that limits viral replication, provides non-specific protection against SARS-CoV-2-induced lung viral burden, and host morbidity in a co-infection mouse model.

Recent M.tb and SARS-CoV-2 mouse co-infection studies (43–45) have similarly observed reduced SARS-CoV-2 infection severity in co-infected animals compared to those infected with SARS-CoV-2 alone. While these studies have offered model insights, the underlying mechanism(s) behind the protective phenotype in co-infected settings remains incompletely understood. In this study, by characterizing a discrete co-infection model using virulent M.tb and variant of high importance and incorporating *in vitro* studies we aimed to uncover the mechanism that could be leading to the observed protection.

## Materials and methods

### Preclinical animal model

Female and Male K18-hACE2 mice [strain: 2B6.Cg-Tg(K18-ACE2)2Prlmn/J], 6–8 weeks of age were purchased from Jackson Laboratory (Bar Harbor, ME). Mice were housed under pathogen-free conditions at Seattle Children’s Research Institute (SCRI) biosafety level 3 animal facility and were handled in accordance with the specific guidelines of SCRI’s Institutional Animal Care and Use Committee (IACUC). Mice were infected with a low dose (50–100 bacteria) aerosol (LDA) of M.tb HN878 using a Glas-Col whole-body exposure chamber (Glas-Col, Terre Haute, IN). Twenty-four hours post challenge the lungs of three mice were homogenized and plated on Mitchison 7H11 agar (Thermo Fisher Scientific, Waltham, MA) to confirm delivery of 50–100 CFU per mouse. For SARS-CoV-2 infection mice were first put under anesthesia with intraperitoneal (i.p.) administration of Ketamine (Patterson Veterinary, Loveland, CO) and Xylazine (Patterson Veterinary). SARS-CoV-2 clinical isolates were administered at 200 PFU via intranasal installation of 40µL per nare. Following SARS-CoV-2 infection mice were weighed daily. Animals that reached 20% weight loss and/or exhibited physical signs of morbidity were humanely euthanized.

### Cells and pathogens

Vero TMPRSS2 (National Institute for Biological Standards and Control (NIBSC), Hertfordshire, England), Vero E6 (ATCC, Manassas, VA), and Calu-3 epithelial cells (ATCC) were maintained at 37 °C + 5% CO_2_ in Dulbecco’s modified Eagle’s medium (DMEM) supplemented with 10% fetal bovine serum (FBS), 2 mM L-glutamine, and 1% penicillin/streptomycin (cDMEM). Cells were tested regularly for mycoplasma with Mycoplasma PCR detection kit (MilliporeSigma, Burlington, MA).

SARS-CoV-2 Beta (hCoV-19/SouthAfrica/KRISP-EC-K005321/2020) was obtained from BEI Resources and housed under standard BSL-3 laboratory conditions. SARS-CoV-2 virus was propagated and titered by plaque assay in Vero E6 cells. Cultured cells were infected with the original stock at a MOI of 0.1 and incubated at 37°C + 5% CO_2_ for 72 h. Supernatants were harvested, centrifuged to remove debris, aliquoted and frozen at −80°C.

### Bacterial counts

At the indicated time points harvested organs were homogenized in DMEM using gentleMACS Octo Tissue Dissociator (Miltenyi, Bergisch Gladbach, Germany). Serial dilutions of organ homogenates were made in PBS with 0.05% Tween80, and aliquots of dilutions were plated on Middlebrook 7H10 agar tri-plates (Molecular Toxicology, Boone, NC), as previously described (46, 47). After 3–4 weeks of incubation at 37°C + 5% CO_2_, colony counts were recorded. Bacterial burden, in colony forming units (CFU) per organ, was calculated, and expressed as Log10.

### Viral load measurements

Viral burden was measured with the plaque forming assay (PFA) using similar techniques described previously (48). Vero TMPRSS2 cells were plated in 6-well plates one day prior to titers at 4.8x10^5^ cells/mL in 2mL of cDMEM per well. Harvested organs were homogenized in DMEM containing 1% FBS (D1 media) using the gentleMACs Octo Dissociator. Organ homogenates were serially diluted ten-fold using D1 media and added dropwise to the plated Vero cells. Plates were incubated at 37 ˚C + 5% CO_2_ for 60 minutes, with 15-minute intervals of rocking plates in all directions. After 60 minutes, 2mL of overlay media comprised of D1 media and 0.2% agarose was added to each well and incubated at 37°C + 5% CO_2_ for 48 hours. Cells were then fixed with 2 mL of 10% Formaldehyde solution and incubated at room temperature for 30 minutes. The overlay was removed, and cells stained with 1mL of Crystal Violet (BD Biosciences, Franklin Lakes, NJ) per well for 20 minutes. Lastly, each well was washed with 1mL of PBS and the number of plaques in each well were recorded.


PFUmL=# of plaques(dilution factor x sample added)

### Histology

At the indicated time points, three whole lung and accessory lobes were collected per group and fixed in 10% Neutral Buffer Formalin (NBF) for 24 hours. The fixed lung samples were embedded in paraffin and sectioned by the University of Washington histology core. Blinded slides were sent to Colorado State University and stained with hematoxylin and eosin (H&E) then analyzed by veterinary pathologist Dr. Brendan Podell as previously published (46, 47). H&E stained sections were scanned at 20X magnification using an Olympus VS120 microscope, Hamamatsu ORCA-R2 camera, and Olympus VS-ASW 2.9 software. Visiopharm software was used for image analysis. For each tissue section, a region of interest (ROI) was generated at a low magnification with a custom tissue detecting algorithm using decision forest training and classification to differentiate tissue versus background based on color and area. Lesions were identified within tissue ROIs at a high magnification with an additional custom-made algorithm using decision forest training and classification based on staining intensity, color normalization and deconvolution, area, and morphological features. Percent lesion calculations were integrated into the same algorithm and calculated from tissue area and lesion area as designated by the ROI and lesions detected. Lesion identification and quantification were then reviewed by Dr. Podell (46, 47).

### Flow cytometry

Cell populations within the lung were measured kinetically utilizing methods previously published (47). Briefly, lung homogenates were incubated in RBC lysis buffer (eBioscience/Thermo Fischer Scientific), washed and resuspended in RPMI 1640 + 10% FBS, and then evenly dispensed into 96-well round bottom plates. Cells were stained for surface markers with fluorochrome-conjugated monoclonal antibodies against mouse Ly6G (FITC, clone 1A8, Biolegend), Ly6C (PerCP-Cy5.5, clone HK1.4, eBioscience), MHCII I-A/I-E (eF450, clone M5/114.15.2, Invitrogen), CD11c (Bv510, clone N418, Biolegend), CD3 (Bv650, clone 17A2, Biolegend), CD19 (APC, clone 6D5, Biolegend), CD11b (Alexa700, clone M1/70, eBioscience), NK1.1 (PE, clone PK136, eBioscience), CD64 (PE-Cy7, clone X54–5/7.1, Biolegend) and 1 μg/mL of Fc receptor block anti-CD16/CD32 (clone 93, eBioscience) in PBS with 1% bovine serum albumin (BSA) for 15 minutes at room temperature. Samples were washed and before removing samples from the BSL3, samples were incubated in 4% paraformaldehyde for 30 minutes. After wash and resuspension in PBS + 1% BSA, cells were acquired on a BD Bioscience LSRII flow cytometer (BD Bioscience) and analyzed using FlowJo version 10.8.1 (BD Bioscience).

### Cytokine measurement

Bronchoalveolar lavage fluid (BALF) was collected by flushing lungs with 1X PBS, then centrifuged at 400g for 7 minutes to remove cellular debris and filtered. The processed BALF was used in the Meso Scale Discovey (MSD) V-PLEX Proinflammatory Panel 1 Mouse kit (#K15048D), V-PLEX Cytokine Panel 1 Mouse Kit (#K15245D) and U-PLEX Interferon Combo 1 (#K15320K) to measure cytokine levels on the MESO QuickPlex SQ 120MM (Meso Scale Diagnostics, Rockville, MD). For similar *in vitro* endpoints from cultured Calu-3 supernatants, an MSD human U-PLEX Viral Combo 1 kit was used (#K15343K-1).

### *In vivo* RT-qPCR

Accessory lung lobes from mice at specified time points post SARS-CoV-2 co-infection were harvested and homogenized in 900µL of Qiazol, followed by RNA extraction using the QIAGEN RNeasy Plus Universal mini kit according to the manufacturer’s protocol (QIAGEN, Hilden, Germany). RNA was eluted into 30μl. RNA concentration and quality was determined using the NanoDrop 8000 (Thermo Fisher Scientific) and stored at -80°C until assayed. The obtained RNA was then utilized in the High-Capacity RNA-to-cDNA kit for cDNA synthesis using SuperScript™ IV Reverse Transcriptase (Thermo Fisher Scientific), containing a reverse transcriptase with a high-fidelity enzyme following manufacture protocol.

For Fluidigm Real-Time PCR and Dynamic Array IFC (Integrated Fluidic Circuit) Setup, specific target amplification (STA) was done as per the manufacturer’s recommendations as the initial step (pre-amplification of cDNA) for the Biomark HD system (Standard BioTools, formerly Fluidigm) carried out on the Standard BioTools 48.48 Gene Expression (GE) Dynamic Array integrated fluidic circuit (IFC) (49). Assay-sets (primers only) were combined as a delta gene multiplex pool (see **Supplementary Table 1**). Preamplification was carried out for each cDNA sample against a reaction-set. Exonuclease I was then used to clean up the preamplification reactions.

Subsequently, the Biomark Chip was primed, and assay premix for each target was aspirated into the IFC assay inlets for a final concentration of 9 µM primers and 2.5 µM probe per reaction, and pre-amplified samples were aspirated into sample inlets. The IFC was then run in the Biomark HD thermocycler, using the manufacturer-supplied thermal cycling conditions. Results were analyzed using the Fluidigm Real-time PCR Analysis software, where thresholds were manually defined, the baseline was automatically assigned, and a Cycle of quantification (Cq) cut-off value of 38 was applied. The cycle threshold (Ct) values for the candidate housekeeping gene, *RPL13*, and target genes were obtained, and delta-delta CT values were calculated.

### *In vitro* experiments

For the *in vitro* experiments, frozen human peripheral blood mononuclear cells (PBMCs) and whole blood were procured from Bloodworks Northwest (Seattle, WA). PBMCs were thawed, counted, and resuspended to a concentration of 2x10^6^ cells/mL, then rested overnight in RPMI media supplemented with 10% FBS and 1% penicillin/streptomycin at 37°C + 5% CO_2_. Cells were counted the next day and viability was assessed before being adjusted to a concentration of 1.5x10^6^ viable cells/mL. Subsequently, the cells were infected with M.tb HN878 at a multiplicity of infection (MOI) of 1 and incubated for 96 hours. Following infection, the cells were centrifuged at 700g for 3 minutes, and the supernatants were collected and filtered through a 0.22-micron filter. Vero or Calu-3 cells were plated and treated with supernatants for 24 hours at 37°C + 5% CO^2^. Media-only treated cells were used as controls. Post-treatment, the cells were challenged with 75 PFU of SARS-CoV-2 Beta, and plaques were recorded 48 hours post-infection using the viral titer PFA described above.

To assess mRNA expression in cultured cells, RNA was extracted from cultured Calu-3 cells using the QIAGEN RNeasy Plus Universal mini kit. The cells were harvested using 900µL of Qiazol, followed by RNA isolation and cDNA synthesis employing the High-Capacity RNA-to-cDNA kit. RNA and cDNA concentration and quality was determined using the NanoDrop 8000 (Thermo Fisher Scientific) and stored at -80°C until assayed. Quantification of mRNA levels was performed using the GoTaq qPCR and RT-qPCR Systems kit from Promega, following the manufacturer’s protocol, and the StepOne Plus Real-Time PCR System (Thermo Fisher Scientific). The mRNA expression levels of Calu-3 cells are presented as Log2 fold change (FC) compared to media-only treated cells and normalized to the housekeeping gene, *Beta Actin*. Primers used were selected from published sequences in PrimerBank (RRID: SCR_006898) (see **Supplementary Table 2**).

### Neutralization assay

For neutralization studies, PBMCs were thawed and counted as described previously. PBMCs were plated in 12-well plates at 2.25x10^6^ cells/mL in one mL of RPMI media supplemented with 10% FBS and 1% penicillin/streptomycin. Neutralizing antibodies for human CD4 (BE0351, BioXcell), Lebanon, NH), CD8α (BE0004–2, BioXcell), CD314 (BE0288, BioXcell), and relevant isotype controls, mouse IgG1(BE0083, BioXcell), and mouse IgG2 (BE0085, BioXcell) were then administered at 10µg/mL, 20µg/mL and 100µg/mL in 1 mL. PBMCs and neutralizing antibodies were incubated for 1 hour at 37°C + 5% CO_2_ prior to infection with M.tb HN878 at MOI of 1 for 96 hours. For IFNγ neutralization, the IFNγ antibody (BE0235, BioXcell) was added directly to the supernatant from PBMCs infected with M.tb HN878 at 10µg/mL, 20µg/mL and 100µg/mL escalating doses. After infection, supernatants were filtered through a 0.22-micron filter. Vero cells were treated with filtered PBMC supernatants for 24 hours, then challenged with 75 PFU of SARS-CoV-2 Beta, and plaques were recorded 48 hours post-infection using the viral titer PFA described above.

### Statistical analysis

Survival analysis was based on the Mantel-Cox log-rank test with Bonferroni correction for multiple comparisons and carried out using GraphPad Prism 9.3.1 (GraphPad Software, San Diego, CA). Bacterial burden, viral titers, cytokine levels, and cell populations (percent frequency and counts) were assessed at a single time point using one-way ANOVA with Tukey’s multiple comparison test to compare between infection groups. Flow cytometry data was assessed using FlowJo v10.8.1 (BD) and statistical analyses were performed using GraphPad Prism 9.3.1 software. The graphics were made with Biorender. Heat maps of *in vivo* mRNA expression were created with RStudio using ‘pheatmap’ function. P values < 0.05 were considered significant and labeled accordingly in each of the figures (* = P<0.05, **= P<0.01, ***=P<0.001, ****= P<0.0001). Outliers were identified using Grubbs’ test at alpha 0.05.

## Results

### Active M.tb infection enhanced host survival and decreased viral burden after SARS-CoV-2 challenge

**Figure 1:**
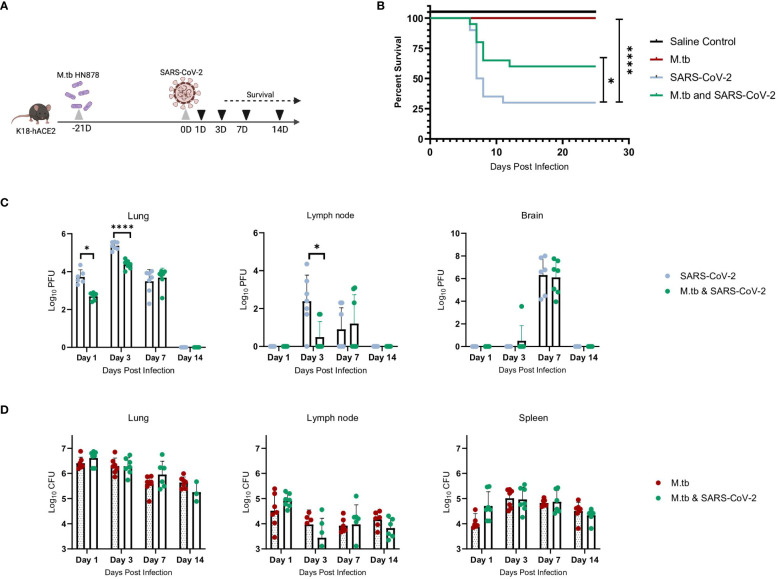
M.tb and SARS-CoV-2 co-infection animal model. **(A)** Experiment scheme for M.tb and SARS-CoV-2 co-infections including selected analysis time points. Image made with Biorender. **(B)** Survival analysis of male and female infection groups with 20 mice per group (10 mice per sex). Mouse weights (n=20/group) were recorded daily, and percent weight change calculated from the maximum recorded weight. (* = P<0.05 and **** = P<0.0001, Mantel-Cox and Wilcoxon). **(C)** Lung, lymph node and brain homogenates from seven female mice per group were used in a plaque formation assay (PFA) to measure viral titers. Each time point analyzed using unpaired T-Test with Welch’s T Test and alpha of 0.05 (* = P<0.05 and **** = P<0.0001). **(D)** Lungs, lymph node and spleen homogenates from seven female mice per group were plated on 7H10 agar triplates to measure bacterial burden.

We hypothesized that infecting mice first with M.tb to induce an active infection and subsequent immune response, followed by co-infection with SARS-CoV-2, could potentially alter disease outcomes and affect survival endpoints. Highly virulent W-Beijing clinical strain, M.tb HN878, was delivered as a low dose aerosol challenge (LDA, 50–100 bacteria) to female and male K18-hACE2 mice. Three weeks post-M.tb infection, mice were challenged with 200 plaque forming units (PFU) of SARS-CoV-2 Beta. (**Figure 1A**). Male and Female mouse cohorts (n=10 per sex) were assessed for survival following infection with M.tb, SARS-CoV-2, or co-infection. Mice singularly infected with SARS-CoV-2 had significantly lower survival rates compared to those in the saline or M.tb infected groups. However, the M.tb and SARS-CoV-2 co-infected group showed a significantly higher survival rate than the group infected solely with SARS-CoV-2 (**Figure 1B**). The increased survival amongst the co-infection group versus the singular SARS-CoV-2 infection group suggests prior M.tb infection may provide partial protection from SARS-CoV-2 challenge, in alignment with our hypothesis.

Viral titers of SARS-CoV-2 were evaluated locally and systemically to determine if co-infected mice have differences in viral load magnitude or distribution. In alignment with prior work (43–45), the co-infected group exhibited decreased lung viral titers at day 1 post-co-infection and significantly decreased lung and lymph node viral titers at day 3 post-co-infection, the anticipated viral peak of our collection timeline, compared to SARS-CoV-2 alone cohorts (**Figure 1C**). There was no difference in viral burden in brain samples when comparing the two infection groups (**Figure 1C**). There was no difference in CFU between the groups for all organs and time points, suggesting the exhibited protection from morbidity was not due to a change in M.tb burden (**Figure 1D**).

**Figure 2:**
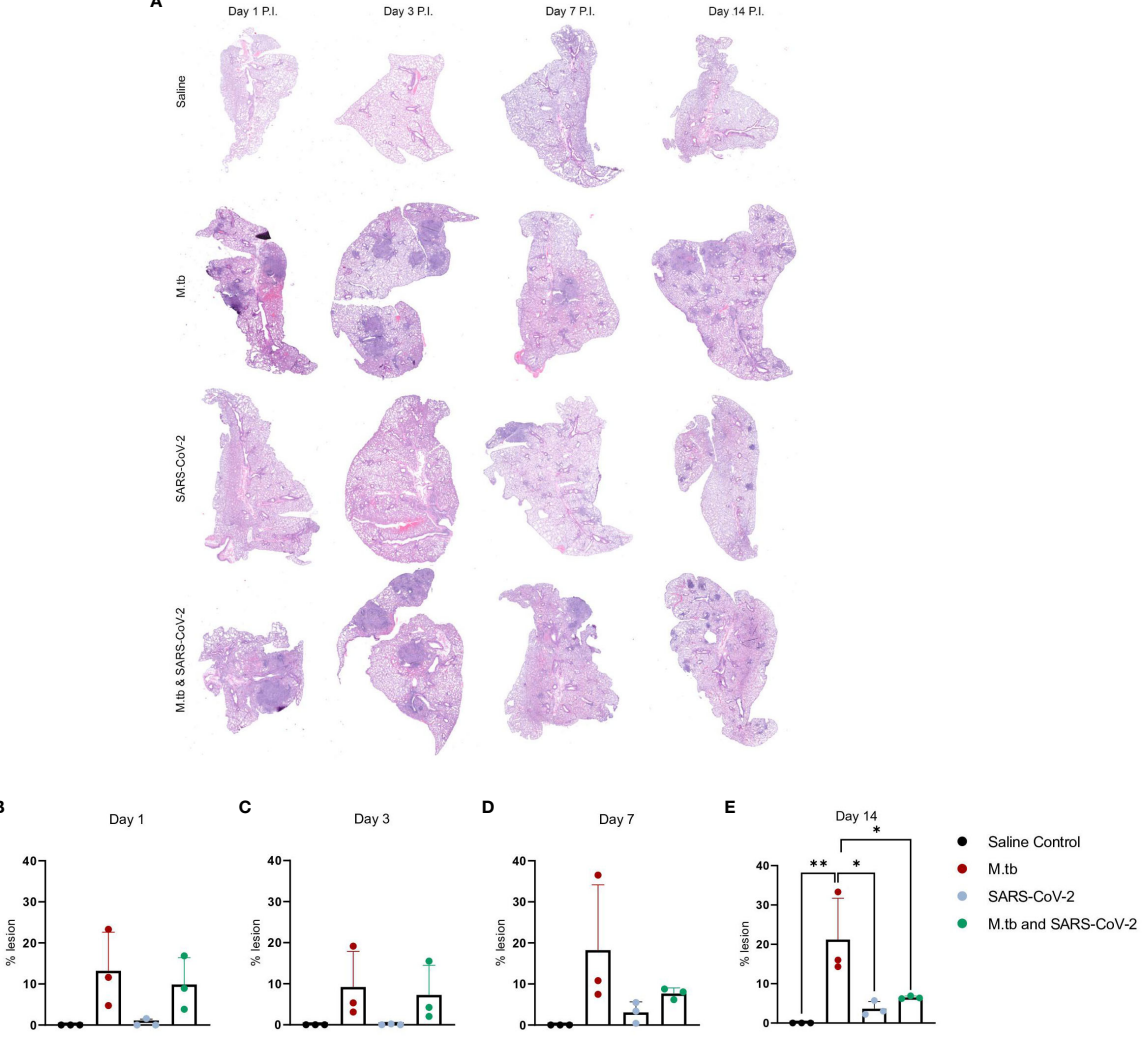
Kinetic quantitative lung histopathology among infection groups. **(A)** Representative H&E images of accessory lung lobe sections showing the presence of pulmonary lesions (dark purple). **(B)** Percent lesion was calculated by dividing the lesion area by the non-lesion area. Each time point was analyzed using one-way ANOVA alpha of 0.05 (* = P<0.05, **= P<0.01).

We have previously seen that bacterial burden can be uncoupled from pulmonary pathology in mouse models of TB, where pulmonary disease and morbidity endpoints may be driven more by host factors (46). Interestingly, when assessing the lung pathology in these co-infection studies (**Figure 2A**) there was no significant difference in percent lesion area between co-infected animals and the SARS-CoV-2-only infected group at day 1 and 3 (**Figures 2B, C**). However, by day 7 there was a trend towards decreased lesion scores in the co-infected groups compared to the M.tb only infection group (**Figure 2D**), which became significant by day 14 (**Figure 2E**). This trend has been published previously (44) and speaks to the complexities of lung pathology in the context of co-infection. Conducting additional analysis which more clearly defines the differences between TB and COVID-19 pathology is worth further exploration. While these data suggest infection with SARS-CoV-2 may help resolve acute lesions from existing M.tb infection (**Figure 2E**), the primary focus of this work is to determine how infection with M.tb establishes an inhospitable pulmonary environment for SARS-CoV-2.

### Established M.tb infection influences lung inflammation during acute SARS-CoV-2 infection

**Figure 3:**
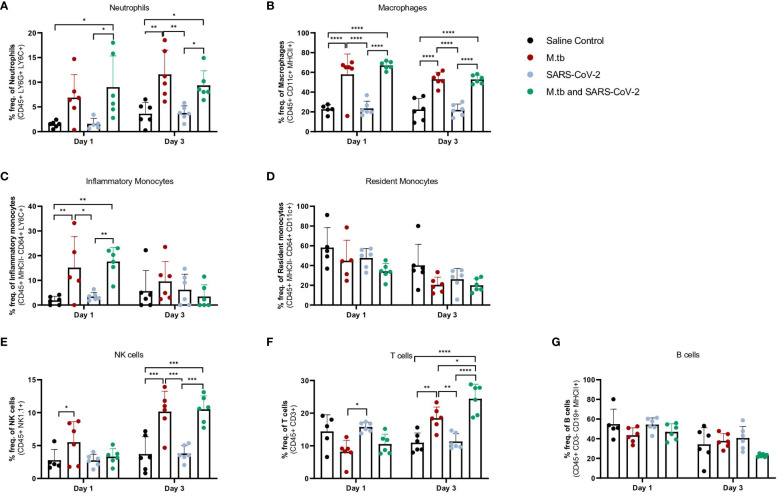
Measured cell populations in mouse lungs following singular infection with M.tb and SARS-CoV-2, and co-infection over time. **(A-G)** Whole lungs from six mice per group per time point were homogenized, processed, and stained for surface markers to measure percent frequency of immune cell populations at 1 day or 3 days following SARS-CoV-2 infection. Significant differences between cohorts at each time point were determined by One-way ANOVA, alpha of 0.05 (* = P<0.05, **= P<0.01, ***=P<0.001, ****=P<0.0001).

**Figure 4:**
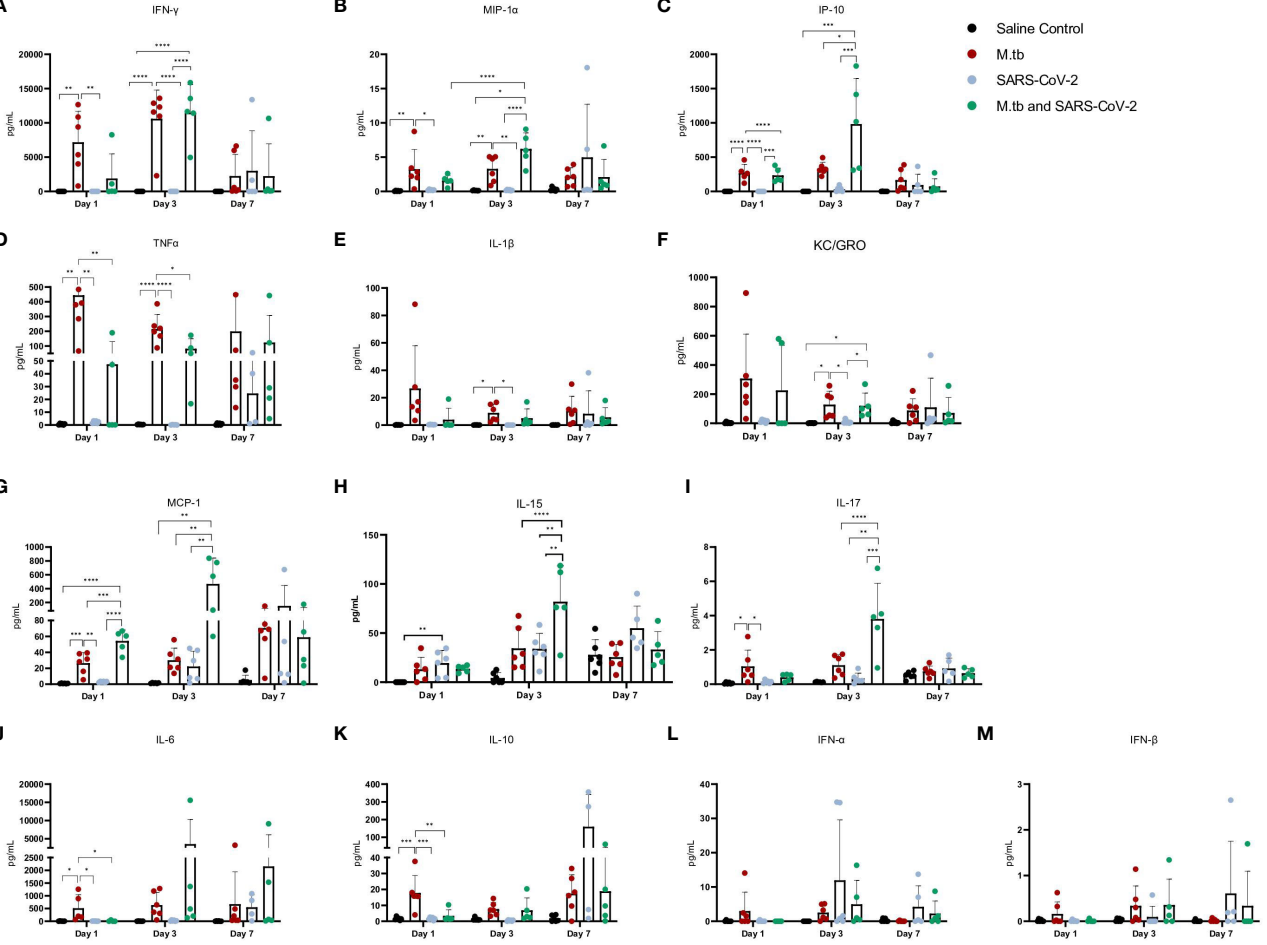
Cytokine and chemokine responses in the lung early after infection with SARS-CoV-2 or co-infection with M.tb. **(A-M)** Bronchoalveolar lavage fluid from seven female mice per group was collected 1, 3, and 7-days post-infection with SARS-CoV-2. Significant differences between cohorts at each time point was determined by one-way ANOVA, alpha of 0.05 (* = P<0.05, **= P<0.01, ***=P<0.001, ****=P<0.0001).

**Figure 5:**
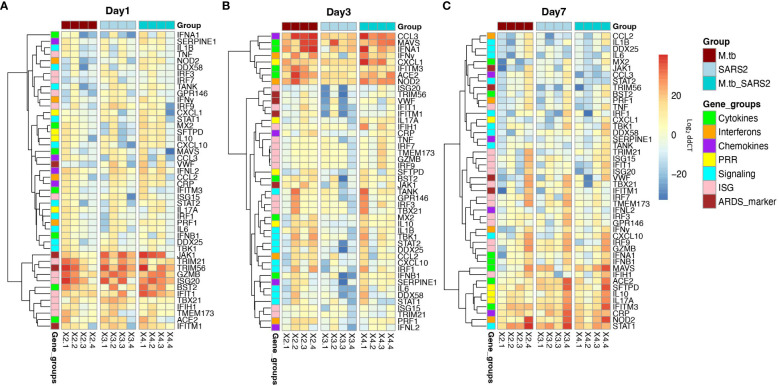
Lung mRNA expression of inflammatory-related genes early after infection with SARS-CoV-2 or co-infection with M.tb. Accessories lobes from four female mice per group were collected 1, 3, and 7-days post-SARS-CoV-2 infection used in RT-qPCR to measure the delta-delta CT values. Heat map depicts Log2 relative expression (delta-delta CT) of selected cytokines, chemokines, interferons, ISGs, and genes involved in inflammatory pathways and lung inflammation **(A)** 1-day post SARS-CoV-2 infection **(B)** 3-days post SARS-CoV-2 infection, and **(C)** 7-days post SARS-CoV-2 infection. Expression was normalized to non-infected saline control mice. Graphs were created using RStudio.

Given the partial protective phenotype displayed by the co-infection model, the immune profiles of M.tb-infected, SARS-CoV-2-infected and co-infected animals were evaluated. Using flow cytometry, the kinetic influx of immune cells to the lung following co-infection were compared to the other cohorts. Both M.tb-infected mice (21 days post infection) and M.tb-infected mice subsequently infected with SARS-CoV-2 (co-infected group) showed elevated levels of neutrophils and macrophages at the day 1 and 3 post-virus challenge time points, and increased influx of T cells and NK cells at day 3 compared to the group infected with SARS-CoV-2 only (**Figure 3**). Absolute cell counts mirroring these trends were also observed (**Supplementary Figure 3**). This showcases the influence chronic M.tb infection has on the inflammatory environment of the lung. Bronchioalveolar lavage fluid (BALF) from M.tb-infected and co-infected mice contained significantly elevated IL-6, TNF-α, IFNγ, IP-10, MIP-1a, MCP-1, and KC-GRO at days 1 and 3 post-co-infection, while the SARS-CoV-2 infection group displayed delayed induction of these same effector molecules until day 7 (**Figure 4**). Interestingly, there was an absence of strong kinetic patterns of increases or persistent decreases in inflammatory gene expression within the lung across infection groups (**Figures 5A-C**). On day 3 there were trends of increased expression in certain ISGs, PRRs and inflammatory pathway genes in M.tb and co-infected groups (**Figure 5B**, **Supplementary Figure 4**).

The increased inflammatory response observed in the co-infected group was initially surprising since the robust pro-inflammatory response has been identified to be detrimental in SARS-CoV-2 infection (13). However, the timing and type of immune response induced may be important for priming the lung to combat SARS-CoV-2 infection. Mechanistic *in vitro* studies were next used to evaluate which innate immune response induced by the primary M.tb infection provide protection against subsequent SARS-CoV-2 infection.

### Cytokines produced from M.tb infected PBMCs provide passive protection from SARS-CoV-2 challenge *in vitro*

To model co-infection *in vitro*, PBMCs were infected with M.tb, as they serve as niche host cells and are responsive to infection. Conversely, SARS-CoV-2 infection was modeled using permissive epithelial cells, which fulfill a similar role. While lung epithelial cells show limited direct responsiveness to M.tb, they exhibit heightened reactivity and transcriptional changes when exposed to M.tb-infected myeloid cells (50). Our investigation aimed to determine whether cytokines generated during initial M.tb infection of immune cells could confer protection against secondary SARS-CoV-2 infection in susceptible bystander epithelial cells.

**Figure 6:**
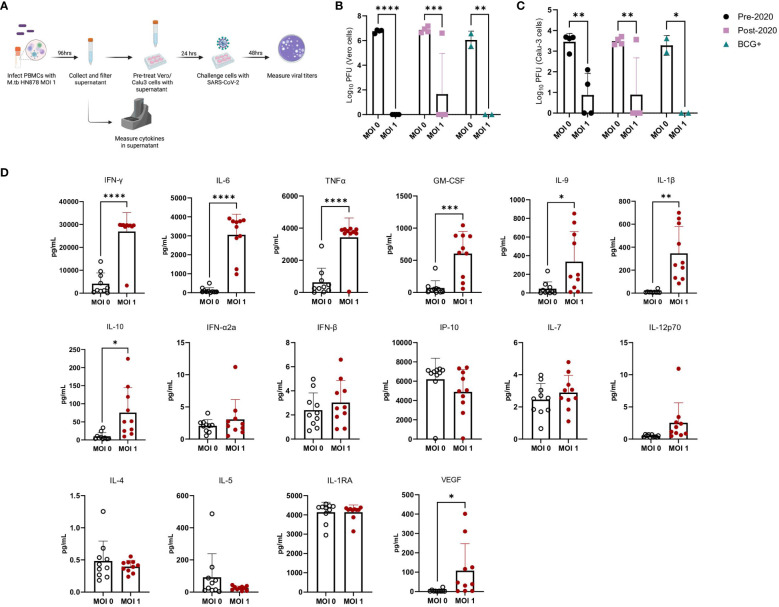
Cytokine levels from M.tb-infected PBMCs and the effect of M.tb infected PBMC supernatants on viral replication in cell culture. **(A)** Experimental scheme of *in vitro* PBMC M.tb infection, **(B)** Viral titers of SARS-CoV-2-challenged Vero cells treated with supernatants from mock-infected or M.tb-infected human PBMCs collected prior to 2020 (N=4), post-2020 (N=4), or from BCG-immunized patients (N=2), and **(C)** viral titers of SARS-CoV-2-challenged Calu-3 cells treated with supernatants from mock-infected or M.tb-infected human PBMCs collected prior to 2020 (N=4), post-2020 (N=4), or from BCG-immunized patients (N=2). Titers between mock-infected and M.tb-infected supernatant treatments for each PBMC group were analyzed using two-way ANOVA (* = P<0.05, **= P<0.01, ***=P<0.001, ****= P<0.0001). **(D)** Cytokine measurements of supernatants from mock-infected or PBMCs infected with M.tb HN878 at a MOI of 1. Measurements analyzed using unpaired T-Test with Welch’s T Test and alpha of 0.05 (* = P<0.05, **= P<0.01, ***=P<0.001, ****= P<0.0001).

We used PBMCs from healthy male and female donors, collected before and after 2019, as well as from Bacille Calmette-Guérin (BCG)-immunized donors (**Supplementary Table 3**), to investigate the effects of prior SARS-CoV-2 exposure or BCG immunization on immune responses. There was additional interest in investigating prior BCG immunizations given the attenuated *M. bovis* vaccine is currently the only licensed TB vaccine and regularly administered in TB endemic regions. While early in the pandemic there were hypotheses that prior BCG immunization may provide protection against SARS-CoV-2 (51), these claims were later dispelled in clinical studies (52, 53). Frozen PBMCs were thawed and either mock-infected or infected with M.tb HN878 at a MOI of 1 for 96 hours (**Figure 6A**). Supernatants were harvested, filtered, and applied to Vero cells, which are highly permissive to SARS-CoV-2 infection, to assess whether cytokines alone could confer protection against SARS-CoV-2 infection. Treatment of Vero cells with supernatants from M.tb-infected PBMCs resulted in significantly reduced viral titers, with no significant differences observed among PBMC groups defined by date or vaccination history (**Figure 6B**). These findings were confirmed using more physiologically relevant Calu-3 human airway epithelial cells (54–56) where diminished viral titers were observed in samples pre-treated with supernatants from M.tb-infected PBMCs (**Figure 6C**). To define the essential elements of protection, the cytokine levels within the supernatants were quantified, revealing increased production of several proinflammatory cytokines, including G-CSF, GM-CSF, TNF-α, IL-1β, IL-6, and IFNγ, following M.tb infection compared to mock-infected PBMCs (**Figure 6D**). Given the absence of significant differences between PBMC groups, subsequent experiments were conducted using PBMCs collected prior to 2019.

**Figure 7:**
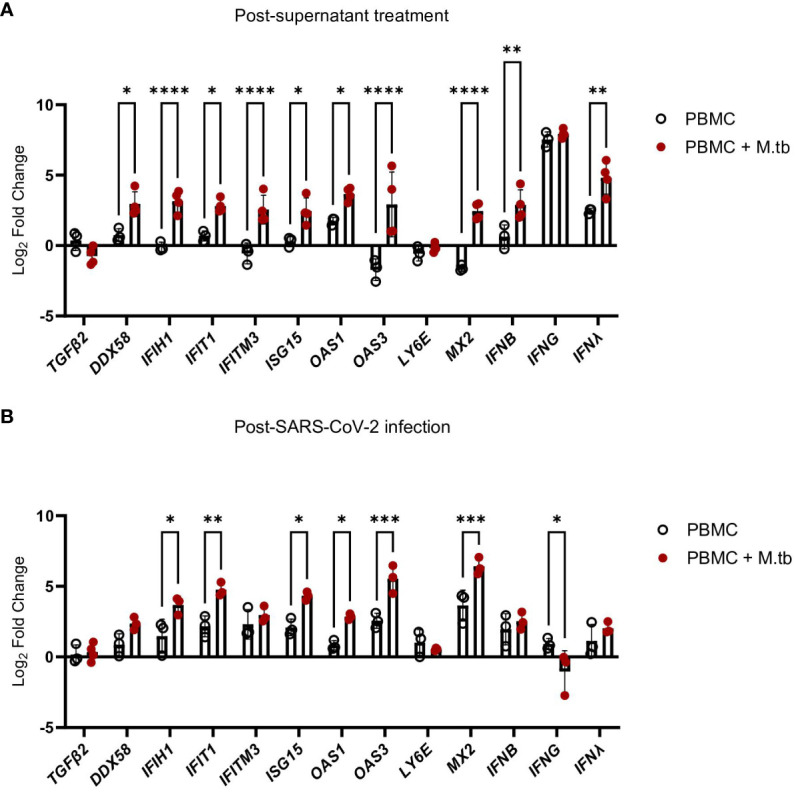
Gene expression changes in Calu-3 epithelial cells treated with supernatants from mock-infected or M.tb-infected human PBMCs and infected with SARS-CoV-2. Graphs depict fold-change expression of ISGs normalized to media-treated cells and the *Beta-Actin* house-keeping gene. **(A)** upregulation of genes 24 hours post-supernatant treatment and **(B)** upregulation of genes 24 hours post-SARS-CoV-2 infection. Expression of genes between mock-infected and M.tb-infected supernatant treatments was analyzed using two-way ANOVA (* = P<0.05, **= P<0.01, ***=P<0.001, ****=P<0.0001).

Supernatant-treated Calu-3 cells were then used in RT-qPCR analysis to determine if cells underwent transcriptional changes upon treatment with supernatants. Treated Calu-3 cells showed significantly increased expression of ISGs such as *OAS1*, *OAS3*, *MX2* and notably, *IFIH1*, the gene encoding MDA5, a primary PRR for SARS-CoV-2, compared to media-treated cells (**Figure 7A**). Subsequently, 24 hours post-SARS-CoV-2 infection, the expression of these ISGs increased in both control and M.tb-infected PBMC supernatant-treated cells, with a significant increase in expression sustained in the supernatant-treated cells (**Figure 7B**). These findings support our hypothesis that prior M.tb infection primes epithelial cells towards resisting viral infection by inducing ISG expression.

### Passive protection from prior M.tb infection restricts viral replication

**Figure 8:**
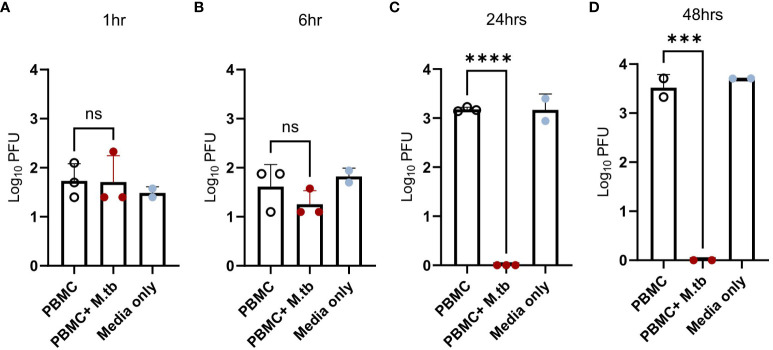
Viral load in Calu-3 cells treated with supernatants from mock-infected or M.tb-infected human PBMCs following infection with SARS-CoV-2. Calu-3 cells were treated with supernatants and infected with 75 PFU of SARS-CoV-2 for **(A)** 1 hour, **(B)** 6 hours, **(C)** 24 hours, and **(D)** 48 hours. Significant differences between groups at each time point was determined by one-way ANOVA, alpha of 0.05 (*** = P<0.001 and **** = P<0.0001).

While pre-treatment led to transcriptional changes and protection from SARS-CoV-2 after 48 hours of infection, pinpointing the stage of the viral infection cycle that may be affected was of interest. To align with our transcriptional data, Calu-3 cells were treated with supernatants or media (positive control) and infected with SARS-CoV-2 for 1, 6, 24, and 48 hours, then assessed for viral load. Following 1 hour of infection, no significant differences in viral load were observed (**Figure 8A**), suggesting no influence or perturbations in viral entry pathways—a result consistent with the expected SARS-CoV-2 doubling time of around 6 hours (57). However, after 6 hours of infection, there was a noticeable trend toward decreased titers in treated Calu-3 cells (**Figure 8B**). By 24 hours, treated cells displayed no plaques likely reaching the limit of detection (**Figure 8C**), suggesting that treated cells were not permissive to replication and actively eliminated the virus, thereby conferring protection.

### Neutralization of IFNγ attenuates protection against SARS-CoV-2

To explore the mechanism of protection, an investigation into the involvement of specific cell types and cytokines was conducted. While type I IFNs are normally associated as the predominant anti-viral response, we did not see significant levels within our measurements. However, we did detect significant levels of IFNγ in both *in vivo* and *in vitro* models, and wanted to determine if blocking IFNγ would attenuate the observed protection. Accordingly, major cell types known to induce IFNγ were targeted.

**Figure 9:**
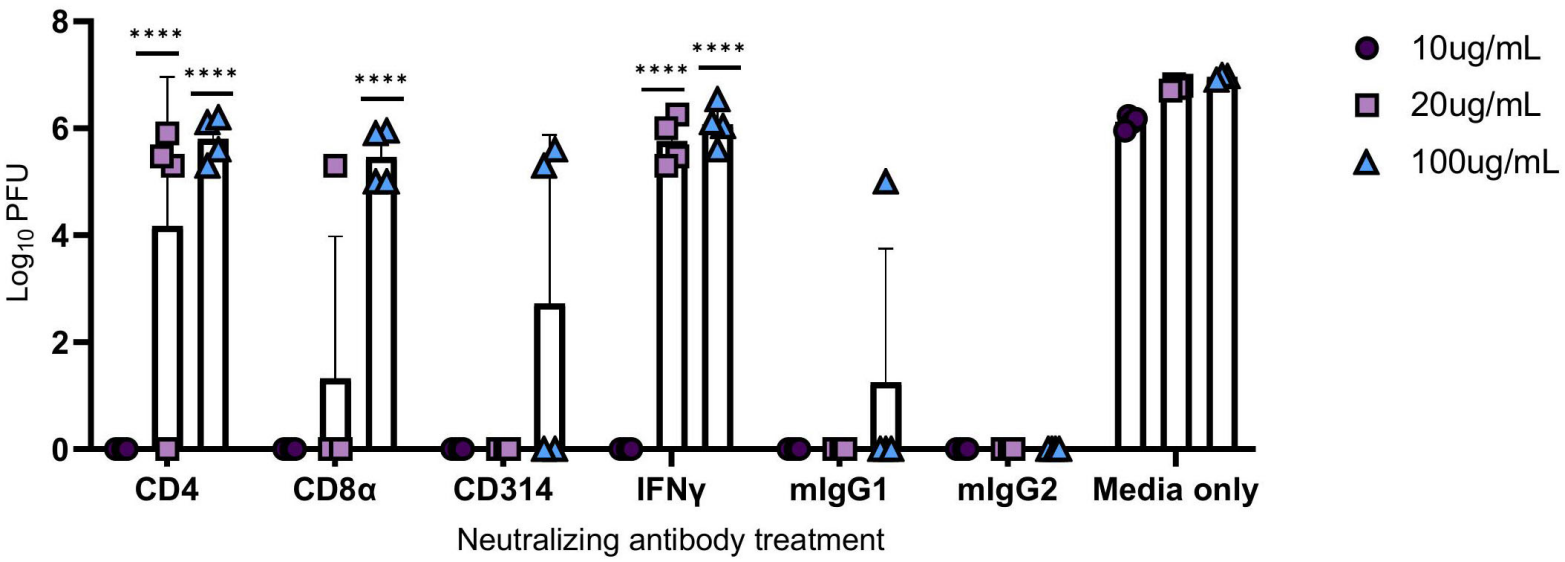
Viral titers following the administration of neutralizing antibodies against immune components. PBMCs were infected with M.tb in the presence of neutralizing antibodies against CD4, CD8α, CD314, and with isotype controls mouse IgG1 (mIgG1) and IgG2 (mIgG2). Some collected supernatants were treated with neutralizing antibodies against IFNγ. Vero cells were treated with the supernatants then challenged with SARS-CoV-2 and PFU recorded 48 hours post-infection. Significant differences of PFUs between neutralization treatments and relative isotype controls, mouse IgG1 (CD4, CD314, and IFNγ) and IgG2 (CD8α) were analyzed using two-way ANOVA (****=P<0.0001). Data are representative of two independent experiments.

Human PBMCs were co-incubated with neutralizing antibodies against CD4+ T cells, CD8α+ T cells, CD314+ NK cells, and IFNγ at increasing concentrations, and then infected with M.tb for 96 hours. Mouse IgG1 and IgG2 antibodies were used as isotype negative controls, while media-only treated cells served as a control for viral replication. After the 96-hour incubation period, supernatants were collected, as previously described, and used to treat permissive Vero cells to measure PFU following SARS-CoV-2 infection. Blocking of CD314+ NK cells did not result in a significant increase in viral titer, and CD8α+ T cells reached significance only at the highest concentration (**Figure 9**). Neutralization of IFNγ led to diminished protection at 20 and 100 μg/mL, as evidenced by an increase in viral titer, highlighting its importance in conferring protection against SARS-CoV-2 (**Figure 9**).

Interestingly, neutralization of CD4+ T cells resulted in an increased viral load with escalating antibody concentrations, suggesting that protection could be dependent on IFNγ and CD4+ T cell activity.

## Discussion

TB and COVID-19 remain leading infectious disease killers, with 1.3 million TB-related deaths reported by the World Health Organization (WHO) in 2022 (58) and a cumulative 6.8 million COVID-19-related deaths as of March 2024 (1). The lack of definitive clinical data on the risks associated with M.tb and SARS-CoV-2 co-infection has sparked significant interest in understanding the interplay between these pathogens. In this study, we contribute to the growing body of data on co-infection using a preclinical model, which allows for the investigation of specific interactions between infections while controlling for factors that influence disease outcomes. This is crucial given the challenges observed in many clinical studies on M.tb and SARS-CoV-2 co-infections, such as issues with study sizes, comorbidities, coinciding risk factors and unknown infection timelines. Consistent with previous findings, we observed a protective effect against SARS-CoV-2 following prior M.tb infection. This model is additive and unique given variations in pathogen strains, including clinical M.tb isolates and variants of interest. While the protective effects of M.tb and SARS-CoV-2 co-infection have been documented, the underlying mechanisms remain largely unknown (43–45). By pairing *in vivo* results with *in vitro* mechanistic studies, we were able to specifically examine the impact of M.tb-induced immune responses on epithelial cells, which are the primary targets of SARS-CoV-2. This focused approach addresses potential limitations of complex *in vivo* systems.

From our studies, we elucidated the importance of IFNγ and CD4+T cell activity in driving the protection seen *in vitro*. An early, and Th1-leaning CD4+ T cell response is deemed important for combatting SARS-CoV-2 (59). Additionally, a study has shown that pre-existing CD4+ T cells induced from previous infection provided protection against SARS-CoV-2 (60). Similarly, IFNγ has demonstrated driving vaccine-induced cellular immunity in K18-hACE2 transgenic B-cell deficient (μMT) mice (61) and recently confirmed to induce early control of SARS-CoV-2 infection when administered intranasally to wildtype C57BL/6 mice (62). Interestingly, clinical studies have reported on M.tb and SARS-CoV-2 co-infected individuals’ limited cellular response to M.tb or SARS-CoV-2 antigens potentially due to anergy or immune exhaustion (11, 63, 64). However, we hypothesize that while prior M.tb induced immune priming can be protective during acute SARS-CoV-2 infection, in certain individuals other factors may hinder this protection, allowing for co-infection to persist and worsening disease outcomes. While we were able to get a controlled look at M.tb and SARS-CoV-2 co-infection in a preclinical model there are many other conditions to consider that may affect co-infection in clinical contexts.

In turn, we remain curious about how our use of a low-dose M.tb infection model, which more closely mimics the chronic stage of human infection, may contribute to the observed protection. Exploring the ultra-low dose M.tb model (65), which delivers 1–3 CFU and strongly mirrors human pathology, may provide insight into whether the diffuse lung immune response exhibited with a low-dose model, or other factors drive protection in co-infection models. Interestingly, it has been reported that the magnitude of viral titers inversely correlated with increasing M.tb infectious dose (29), providing further evidence towards the need of a diffuse infection and accompanied response. Additionally, LMICs with large TB burden are heavily associated with comorbidities that affect TB and COVID-19 severity (66–68). In order to further understand and close the gap between preclinical and clinical studies investigating these additional factors such as sex, metabolic diseases, age, HIV co-infections, and antibiotic resistant M.tb strains in the pre-clinical model, will be vital for furthering knowledge on M.tb and SARS-COV-2 co-infections. Additionally, we acknowledge that differences based on the phase of M.tb infection, such as active versus latent infection, can impact the outcomes of co-infection with SARS-CoV-2, thus warranting further investigation.

Moreover, our study underscores the importance of innate immune induction in protection against SARS-CoV-2. While increased global vaccination has significantly impacted the trajectory and harm of COVID-19, the emergence of humoral immune evasion by SARS-CoV-2 variants of concern highlighted the need for more comprehensive vaccine-induced responses. Our findings further emphasize the crucial role of innate immune responses in combating the earliest stages of viral infections. Additionally, this highlights the need to fine-tune inflammatory responses to ensure they contribute to protection rather than exacerbate detrimental effects. These models help winnow down potential therapeutic targets and define features desirable for prophylactic vaccine strategies.

## Data availability statement

The original contributions presented in the study are included in the article/**Supplementary Material**. Further inquiries can be directed to the corresponding author.

## Ethics statement

Ethical approval was not required for the studies on humans in accordance with the local legislation and institutional requirements because only commercially available established cell lines were used. The animal study was approved by Seattle Children’s Research Institute IACUC. The study was conducted in accordance with the local legislation and institutional requirements.

## Author contributions

BW: Writing – review & editing, Writing – original draft, Visualization, Project administration, Methodology, Investigation, Formal analysis, Data curation, Conceptualization. DF: Writing – review & editing, Methodology, Investigation, Data curation. HA: Writing – review & editing, Methodology, Investigation, Formal analysis, Data curation. BB: Writing – review & editing, Supervision, Methodology, Investigation, Conceptualization. BP: Writing – review & editing, Formal analysis, Data curation. SL: Supervision, Methodology, Writing – review & editing, Project administration, Conceptualization. SB: Visualization, Supervision, Resources, Project administration, Funding acquisition, Conceptualization, Writing – review & editing. RC: Writing – review & editing, Supervision, Resources, Project administration, Funding acquisition, Conceptualization.
