# Supplementary material for: Protective interplay: Mycobacterium tuberculosis diminishes SARS-CoV-2 severity through innate immune priming
Source: Front Immunol. 2024 Jun 20;15:1424374. doi: 10.3389/fimmu.2024.1424374 (PMC11222399; doi:10.3389/fimmu.2024.1424374)
Supplement: Supplementary file 1 [file Table_1.docx]

| Supplementary Table 1. Targets used for *in vivo* RT-PCR | | |
| --- | --- | --- |
| **INDEX** | **Target** | **RefSeq** |
| 1 | *IFNB ​* | NM_010510.2 |
| 2 | *IFNG ​* | NM_008337.4 |
| 3 | *IL-1B ​* | NM_008361.4 |
| 4 | *IL-6* | NM_031168.2 |
| 5 | *IL-17a* | NM_010552.3 |
| 6 | *IL-10 ​* | NM_010548.2 |
| 7 | *IP-10 (CXCL10)* | NM_021274.2 |
| 8 | *RIG-I ​* | NM_172689.3 |
| 9 | *PERFORIN* | NM_011073.3 |
| 10 | *NOD2 ​* | NM_145857.2 |
| 11 | *CRP* | NM_007768.4 |
| 12 | *SP-D* | NM_009160.2 |
| 13 | *IL-28a* | NM_001024673.2 |
| 14 | *ACE2* | NM_001130513.1 |
| 15 | *SERPINE1* | NM_008871.2 |
| 16 | *vWF* | NM_011708.4 |
| 17 | *GAPDH* | NM_001411843.1 |
| 18 | *IFNA* | NM_010502.2 |
| 19 | *IRF7 ​* | NM_001252601.1 |
| 20 | *IRF1* | NM_001159396.1 |
| 21 | *GPR146* | NM_030258.5 |
| 22 | *DDX25* | NM_013932.4 |
| 23 | *MX2* | NM_013606.1 |
| 24 | *ISG20* | NM_001291221.1 |
| 25 | *IFIH1* | NM_001164477.1 |
| 26 | *IFITM1* | NM_001360727.1 |
| 27 | *IRF3* | NM_016849.4 |
| 28 | *JAK1* | NM_013567.1 |
| 29 | *ISG15* | NM_015783.3 |
| 30 | *GRANZYME B* | NM_013542.3 |
| 31 | *BST2* | NM_198095.3 |
| 32 | *TANK* | NM_001378922.1 |
| 33 | *IFIT1* | NM_008331.3 |
| 34 | *IFITM3* | NM_025378.2 |
| 35 | *STAT1* | NM_001357627.1 |
| 36 | *STAT2* | NM_019963.2 |
| 37 | *IRF9* | NM_001159418.1 |
| 38 | *TBK1* | NM_019786.4 |
| 39 | *TRIM 21* | NM_001082552.2 |
| 40 | *TMEM173* | NM_001289591.1 |
| 41 | *TRIM56* | NM_201373.4 |
| 42 | *MAVS* | NM_001206382.1 |
| 43 | *BETA ACTIN* | NM_007393.5 |
| 44 | *TBX21* | NM_019507.2 |
| 45 | *KC-GRO/CXCL10* | NM_021274.2 |
| 46 | *MIP-1a/CCL3* | NM_011337.2 |
| 47 | *MCP-1/CCL2* | NM_011333.3 |
| 48 | *TNF* | NM_001278601.1 |

| Supplementary Table 2. Primers used for *in vitro* RT-PCR | | | |
| --- | --- | --- | --- |
| **Name** | **Forward primer** | **Reverse primer** | **REF** |
| *BETA ACTIN* | CATGTACGTTGCTATCCAGGC | CTCCTTAATGTCACGCACGAT | PB: 4501885a1 |
| *BMP4* | ATGATTCCTGGTAACCGAATGC | CCCCGTCTCAGGTATCAAACT | PB: 157276592c1 |
| *BST2* | CACACTGTGATGGCCCTAATG | GTCCGCGATTCTCACGCTT | PB: 7262372c1 |
| *CTGF* | CAGCATGGACGTTCGTCTG | AACCACGGTTTGGTCCTTGG | PB: 98986335c1 |
| *CXCL10* | GTGGCATTCAAGGAGTACCTC | TGATGGCCTTCGATTCTGGATT | PB: 323422857c1 |
| *dACE2* | GGAAGCAGGCTGGGACAAA | AGCTGTCAGGAAGTCGTCCATT | ref |
| *DDX58* | CTGGACCCTACCTACATCCTG | GGCATCCAAAAAGCCACGG | PB: 77732514c1 |
| *GAPDH* | GGAGCGAGATCCCTCCAAAAT | GGCTGTTGTCATACTTCTCATGG | PB: 378404907c1 |
| *IFIH1* | TCGAATGGGTATTCCACAGACG | GTGGCGACTGTCCTCTGAA | PB: 27886567c1 |
| *IFIT1* | TTGATGACGATGAAATGCCTGA | CAGGTCACCAGACTCCTCAC | PB: 116534936c1 |
| *IFITM3* | GCTG ATCTTCCAGGCCTATG | GATACAGGACTCGGCTCCGG | PMID: 26565900 |
| *IFNB* | ATGACCAACAAGTGTCTCCTCC | GGAATCCAAGCAAGTTGTAGCTC | PB: 50593016c1 |
| *IFNG* | TCGGTAACTGACTTGAATGTCCA | TCGCTTCCCTGTTTTAGCTGC | PB: 56786137c1 |
| *IFN lambda (IL-28B)* | TAAGAGGGCCAAAGATGCCTT | CTGGTCCAAGACATCCCCC | PB: 28144901a1 |
| *ISG15* | CGCAGATCACCCAGAAGATCG | TTCGTCGCATTTGTCCACCA | PB: 193083170c1 |
| *LY6E* | CAGCTCGCTGATGTGCTTCT | CAGACACAGTCACGCAGTAGT | PB: 187827163c1 |
| *MX2* | CAGAGGCAGCGGAATCGTAA | TGAAGCTCTAGCTCGGTGTTC | PB: 11342663c1 |
| *NOD2* | TGGTTCAGCCTCTCACGATGA | CAGGACACTCTCGAAGCCTT | PB: 11545911c1 |
| *OAS1* | TGTCCAAGGTGGTAAAGGGTG | CCGGCGATTTAACTGATCCTG | PB: 74229012c1 |
| *OAS3* | GAAGGAGTTCGTAGAGAAGGCG | CCCTTGACAGTTTTCAGCACC | PB: 45007006c1 |
| *TBK1* | TGGGTGGAATGAATCATCTACGA | GCTGCACCAAAATCTGTGAGT | PB: 309747068c1 |
| *TGFB1* | GGCCAGATCCTGTCCAAGC | GTGGGTTTCCACCATTAGCAC | PB: 260655621c1 |
| *TGFB2* | CAGCACACTCGATATGGACCA | CCTCGGGCTCAGGATAGTCT | PB: 305682568c1 |
| *TMPRSS2* | GTCCCCACTGTCTACGAGG | CAGACGACGGGGTTGGAAG | PB: 227499989c1 |
| *STAT2* | CCAGCTTTACTCGCACAGC | AGCCTTGGAATCATCACTCCC | PB: 291219923c1 |

| **Supplementary Table 3. Patient information from collected human PBMCs** | | | | |
| --- | --- | --- | --- | --- |
| **ID** | **Sex** | **Age** | **Collection Date** | **Other** |
| 5288BW | M | 52 | 12-8-2021 |  |
| 4772BW | M | 30 | 8-19-2021 | CMV pos, |
| 3092BW | F | 62 | 10-6-2021 |  |
| 2698BW | F | 40 | 3-3-2021 | CMV pos, |
| 3253BW | F | 37 | 9-30-2016 |  |
| 2635BW | F | 45 | 8-22-2019 | CMV neg, |
| 2206BW | M | 25 | 5-4-2015 |  |
| 1957BW | M | 27 | 7-6-2017 | CMV neg |
| 1982BW | M | 27 | 11-28-2023 | BCG+ |
| 2735BW | M | 31 | 11-28-2023 | BCG+ |
