# Supplementary material for: Protective interplay: Mycobacterium tuberculosis diminishes SARS-CoV-2 severity through innate immune priming
Source: Front Immunol. 2024 Jun 20;15:1424374. doi: 10.3389/fimmu.2024.1424374 (PMC11222399; doi:10.3389/fimmu.2024.1424374)
Supplement: Supplementary file 2 [file Presentation_1.pptx]

## Slide 1
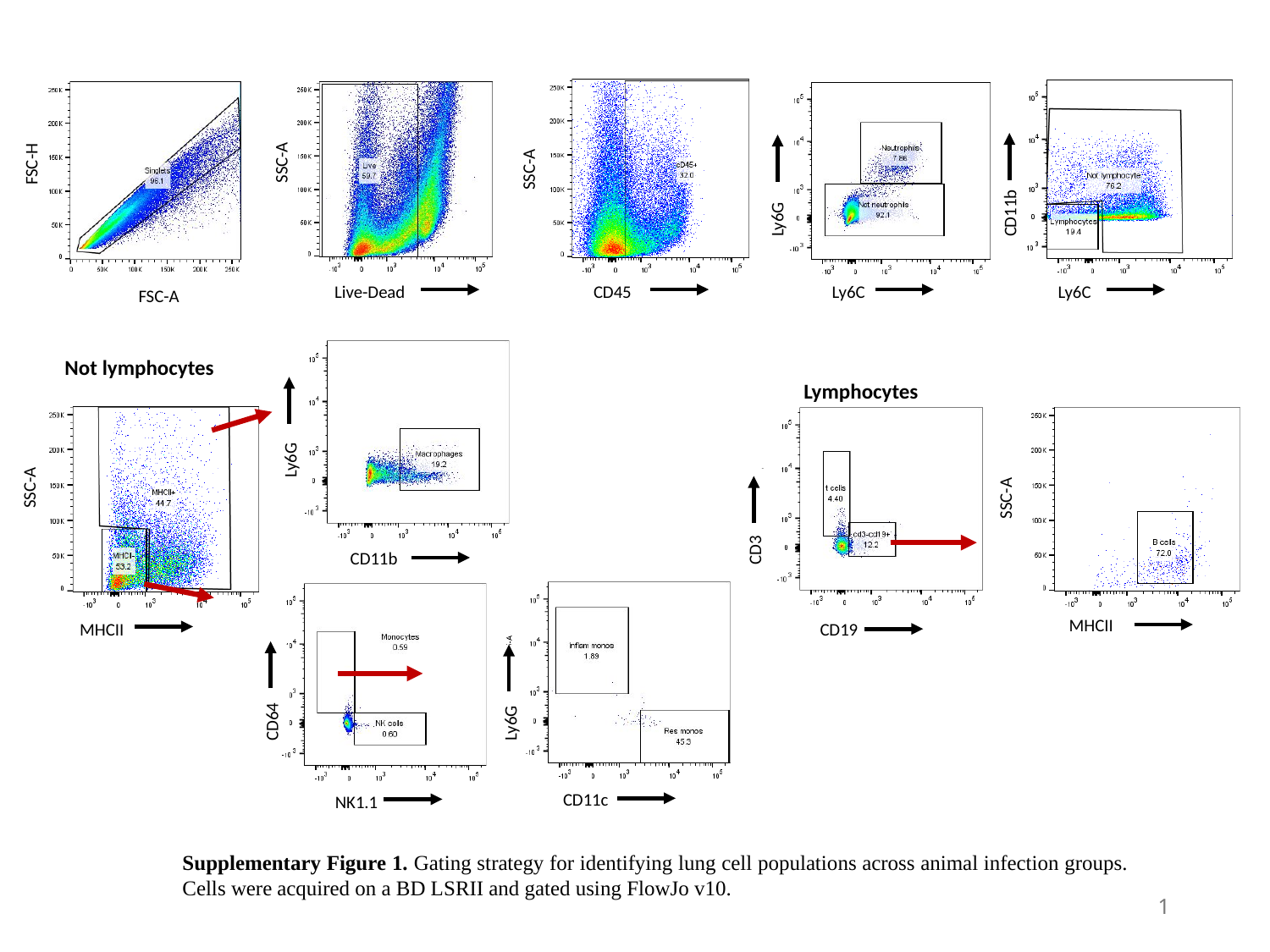

SSC-A
FSC-H
SSC-A
Ly6G
CD11b
Live-Dead
CD45
Ly6C
Ly6C
FSC-A
Not lymphocytes
Lymphocytes
Ly6G
SSC-A
SSC-A
CD3
CD11b
MHCII
MHCII
CD19
CD64
Ly6G
CD11c
NK1.1
Supplementary Figure 1. Gating strategy for identifying lung cell populations across animal infection groups. Cells were acquired on a BD LSRII and gated using FlowJo v10.
1

## Slide 2
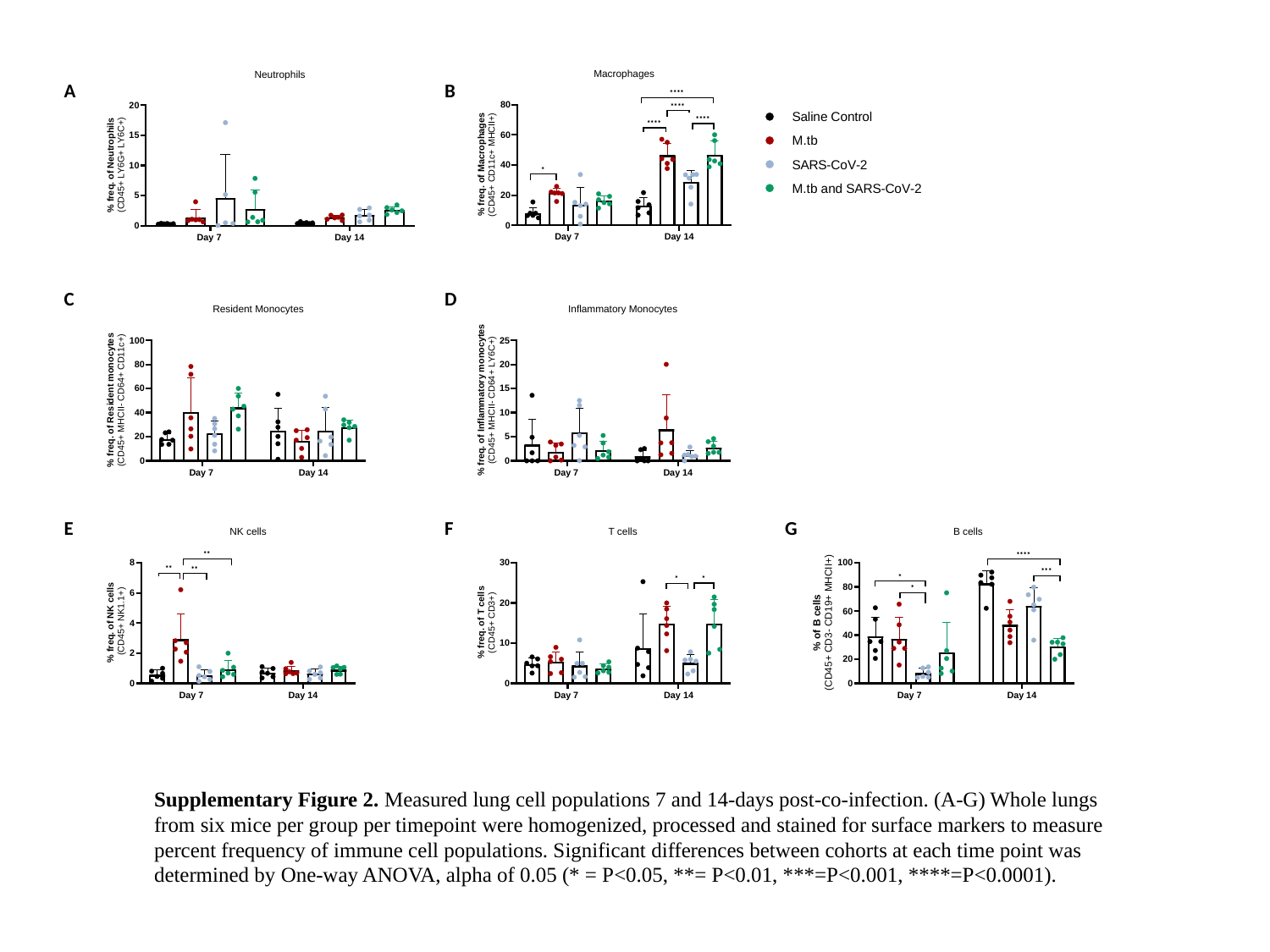

A
B
C
D
G
E
F
Supplementary Figure 2. Measured lung cell populations 7 and 14-days post-co-infection. (A-G) Whole lungs from six mice per group per timepoint were homogenized, processed and stained for surface markers to measure percent frequency of immune cell populations. Significant differences between cohorts at each time point was determined by One-way ANOVA, alpha of 0.05 (* = P<0.05, **= P<0.01, ***=P<0.001, ****=P<0.0001).

## Slide 3
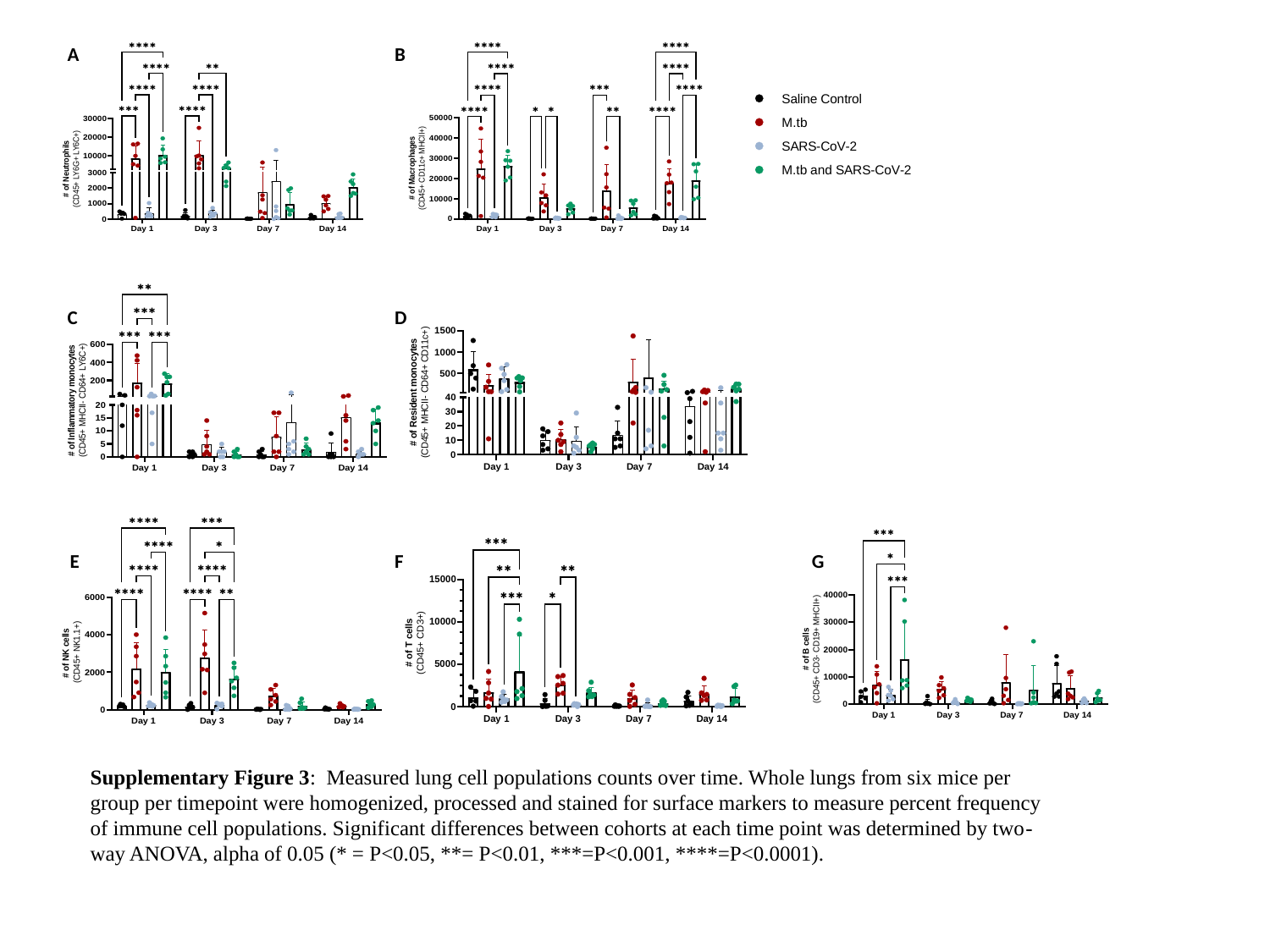

A
B
C
D
E
F
G
Supplementary Figure 3: Measured lung cell populations counts over time. Whole lungs from six mice per group per timepoint were homogenized, processed and stained for surface markers to measure percent frequency of immune cell populations. Significant differences between cohorts at each time point was determined by two-way ANOVA, alpha of 0.05 (* = P<0.05, **= P<0.01, ***=P<0.001, ****=P<0.0001).

## Slide 4
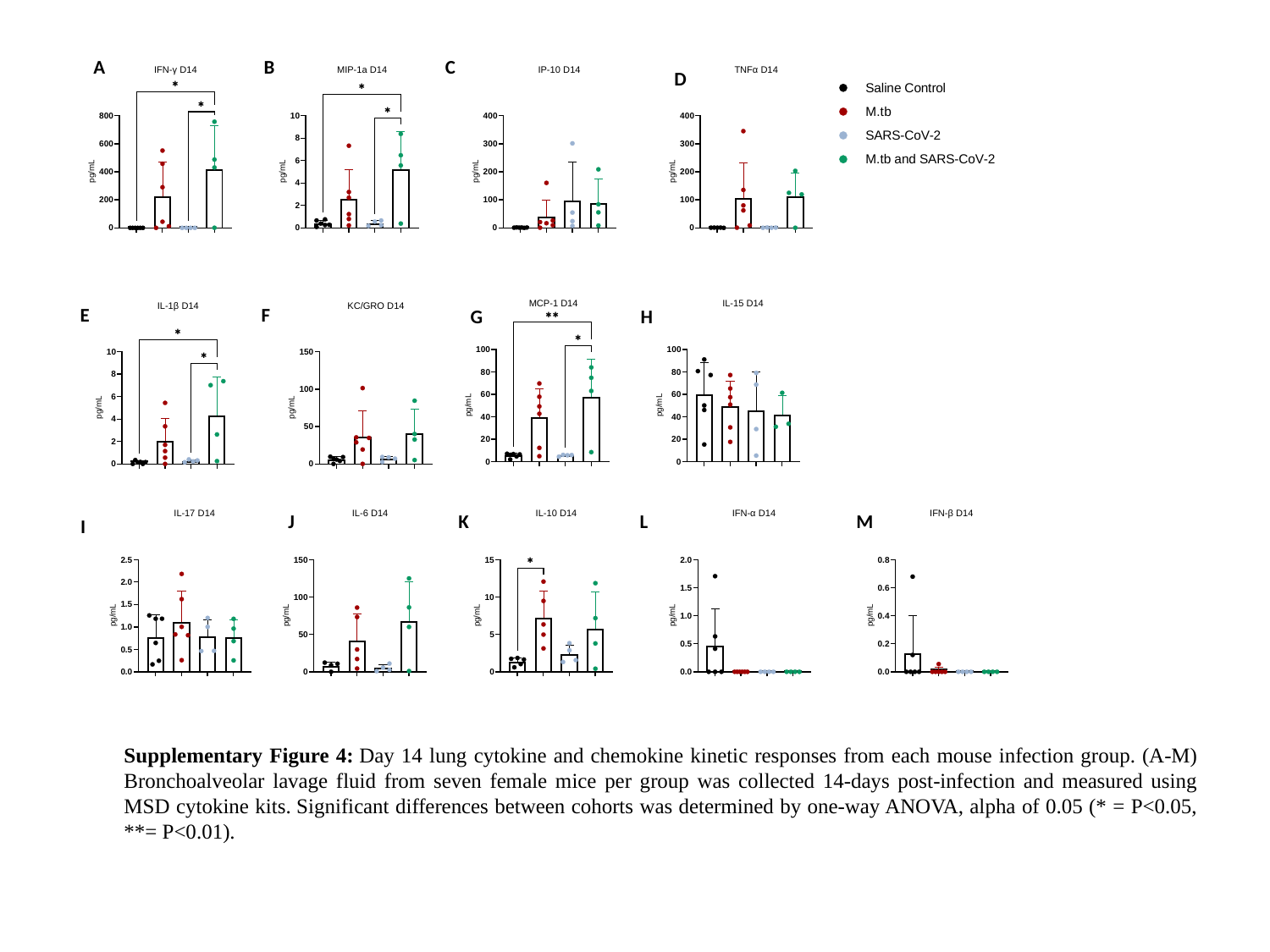

A
B
C
D
E
F
G
H
J
K
L
M
I
Supplementary Figure 4: Day 14 lung cytokine and chemokine kinetic responses from each mouse infection group. (A-M) Bronchoalveolar lavage fluid from seven female mice per group was collected 14-days post-infection and measured using MSD cytokine kits. Significant differences between cohorts was determined by one-way ANOVA, alpha of 0.05 (* = P<0.05, **= P<0.01).

## Slide 5
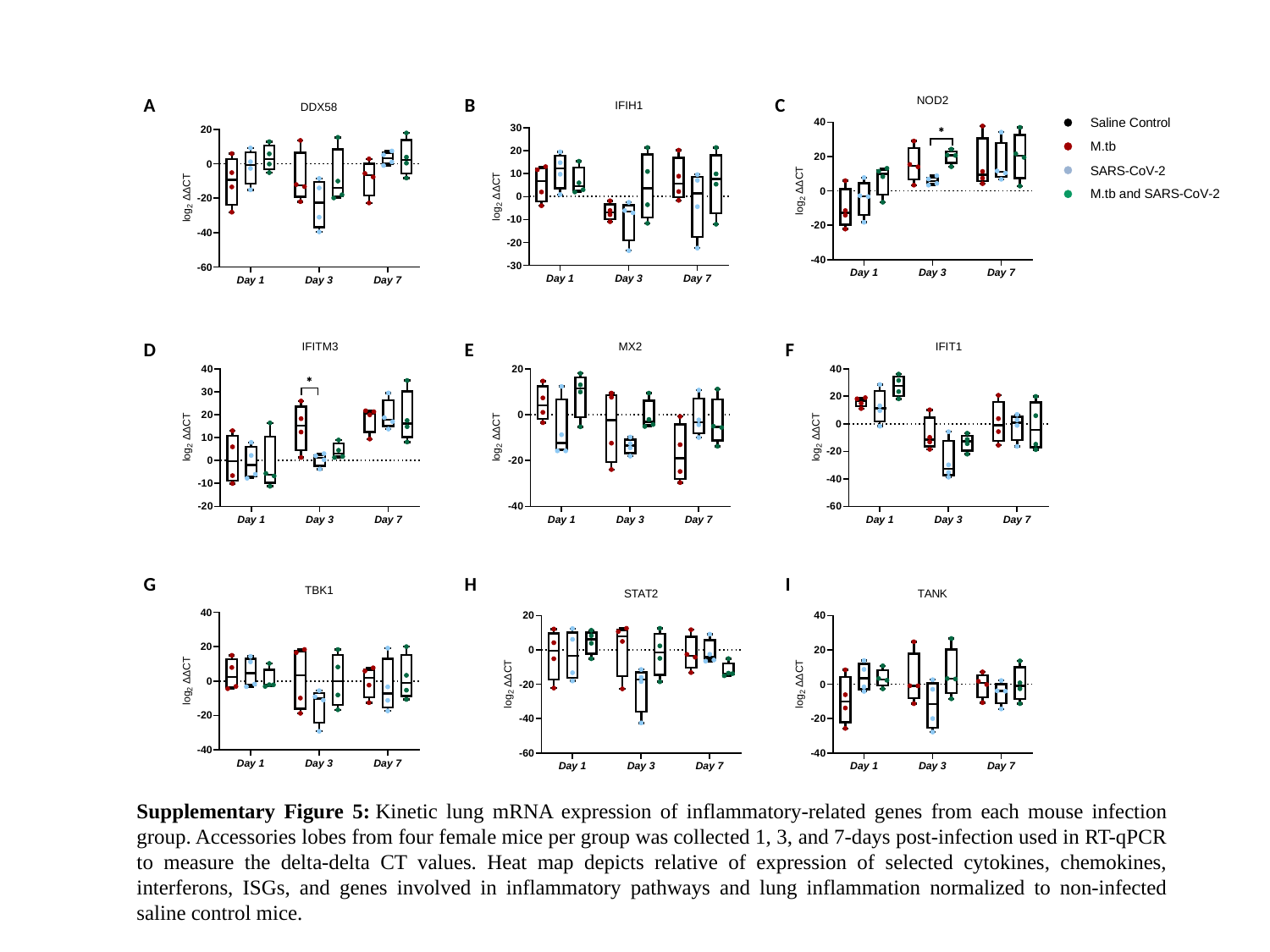

A
B
C
D
E
F
G
H
I
Supplementary Figure 5: Kinetic lung mRNA expression of inflammatory-related genes from each mouse infection group. Accessories lobes from four female mice per group was collected 1, 3, and 7-days post-infection used in RT-qPCR to measure the delta-delta CT values. Heat map depicts relative of expression of selected cytokines, chemokines, interferons, ISGs, and genes involved in inflammatory pathways and lung inflammation normalized to non-infected saline control mice.
